# Cell Density Effects in Different Cell Culture Media and Their Impact on the Propagation of Foot-And-Mouth Disease Virus

**DOI:** 10.3390/v11060511

**Published:** 2019-06-04

**Authors:** Veronika Dill, Janike Ehret, Aline Zimmer, Martin Beer, Michael Eschbaumer

**Affiliations:** 1 Institute of Diagnostic Virology, Friedrich-Loeffler-Institut, Südufer 10, 17493 Greifswald–Insel Riems, Germany; veronika.dill@fli.de (V.D.); martin.beer@fli.de (M.B.); 2 Merck KGaA, Merck Life Sciences, Upstream R&D, Frankfurter Straße 250, 64293 Darmstadt, Germany; janike.ehret@merckgroup.com (J.E.); aline.zimmer@merckgroup.com (A.Z.)

**Keywords:** foot-and-mouth disease virus, cell density, animal-component-free media, chemically defined media, antifoam, suspension cells

## Abstract

Foot-and-mouth disease virus (FMDV) is endemic in many parts of the world. Vaccination is an important control measure, limits viral spread, and can help to eradicate the disease. However, vaccination programs are cost-intensive because of the short shelf life of vaccines and the need for frequent re-vaccination. Animal-component-free (ACF) or chemically defined media (CDM) at high cell densities are a promising approach for the production of inexpensive high-quality vaccines, but the occurrence of cell density effects has been reported for various virus-cell systems in vaccine production. For FMDV, the use of CDM or ACF media for vaccine production has not been studied and no information about cell density effects is available. This work describes the propagation of FMDV in ACF or in CDM. Cells were grown at increasing cell densities and either 100% media exchange or addition of 30% fresh media was performed before infection with FMDV. Increasing cell densities reduced the viral titer and increased yield variability in all media except BHK300G. This effect can be mitigated by performing a 100% media exchange before infection or when using the controlled environment of a bioreactor. The media composition and also a fragile relationship between virus and cell metabolism seem to be causal for that phenomenon.

## 1. Introduction

Foot-and-mouth disease (FMD), caused by an aphthovirus of the family *Picornaviridae* (FMD virus, FMDV), remains a threat to industrial and developing countries alike for diverse reasons. Europe has been severely affected by this disease until late into the second half of the 20th century when it was successfully eradicated through vaccination campaigns and culling of infected livestock [1]. FMD-free countries face extensive monetary losses and economic damages due to trade restrictions and intensive culling in the case of an introduction of FMD [2], while Africa, Asia and the Middle East still suffer from its endemic occurrence [3]. In these countries, FMD threatens the livelihood of farmers, independent of farm size due to losses in milk and meat production as well as the death of young animals [4]. Vaccination campaigns, supported by the Food and Agriculture Organization of the United Nations (FAO) and the World Organisation for Animal Health (OIE), are an important tool to eradicate FMD in endemic areas. Suitable vaccines prevent clinical disease and reduce viral spread [5]. Unfortunately, such programs can become quite cost-intensive, especially due to the short shelf life of the vaccine and the necessity to re-vaccinate every four to twelve months [5]. Therefore, it is essential to produce high-quality vaccines at low cost. FMD vaccines are traditionally produced in batches by growing BHK-21 suspension cells in serum-containing nutrient medium until they reach the desired cell density [4,6]. Cells are then allowed to settle or are centrifuged to remove the spent cell culture media and to resuspend the cells in fresh serum-free media or in media with reduced serum content before viral infection [6]. A promising approach, therefore, are animal-component-free (ACF) or even fully chemically defined media (CDM) for the cultivation of cells in the course of vaccine production. These media minimize not only lot-to-lot variations of poorly defined components such as serum or animal tissue hydrolysates, they also prevent contamination of the product with adventitious viruses, mycoplasmal bacteria or prions [4,7]. Furthermore, higher cell densities can increase the viral yield per run and reduce the cost per dose of vaccine by a more efficient use of bioreactor capacity [8]. Production systems without cell retention usually can perform runs at a total cell density between 1 × 10^6^ and 5 × 10^6^ mL^−1^ for a standard animal-cell bioreactor [9]. However, so-called cell density effects, i.e., cell-specific viral yields that are lower than expected proportional to the cell density in the process [10], have been reported for various virus production systems [8,11,12,13]. While the accumulation of inhibitory factors or a limitation of nutrients cause this decrease in some cases [10,11,14], in others the reasons are still unclear [8,15]. To our knowledge, no information about cell density effects and their possible causes is available for the production of FMDV antigen. In addition, very few studies are available that examine process optimization in FMD vaccine production with chemically defined cell culture media or even animal-component-free media. 

This work describes the propagation of FMDV in a commercially available ACF media and prototypes of CDM at different cell densities in spin tubes as well as in a stirred-tank bioreactor. Possible causes for the loss of viral yield with increased cell density are evaluated and potential strategies for process optimization in vaccine production are discussed. 

## 2. Materials and Methods

### 2.1. Cells and Cell Culture Media

The suspension cell line BHK21C13-2P (in short: BHK-2P; originally derived from the European Collection of Authenticated Cell Cultures specimen 84111301) was adapted to grow in three different cell culture media: the ACF medium Cellvento™ BHK-200 (in short: BHK200) as well as in BHK300B and BHK300G, two prototypes of chemically derived cell culture media (Merck KGaA, Darmstadt, Germany). Cells were maintained in TubeSpin bioreactors (TPP Techno Plastic Products AG, Trasadingen, Switzerland) in a shaker incubator at 320 revolutions per minute (rpm) at 37 °C, 5% CO_2_ and 80% relative humidity. The viable cell density (VCD) and percent viability were evaluated by a dye exclusion method with a TC20 Automated Cell Counter (Bio-Rad, Hercules, CA, USA).

For virus titrations, the adherent BHK21 clone “Tübingen” (in short: BHK164, specimen CCLV-RIE 164 in the Collection of Cell Lines in Veterinary Medicine, Friedrich-Loeffler-Institut [FLI], Greifswald, Germany) was cultured in Minimum Essential Medium Eagle, supplemented with Hanks’ and Earle’s salts (Sigma-Aldrich, St. Louis, MO, USA) with 5% fetal bovine serum. The adherent cells were incubated at 37 °C and 5% CO_2_.

### 2.2. Viruses and Virus Titrations

A laboratory strain of FMDV (A_24_ Cruzeiro, originally isolated in Brazil in 1955) [16] and the more recent isolate O SAU/18/2015 were selected from archival stocks at the FLI. Their origin and passage history can be found in Supplementary Table S1. Viral titers were estimated by endpoint titration with the Spearman-Kärber method [17,18] and expressed as 50% tissue culture infectious dose (TCID_50_) per mL. Virus titrations were performed on the adherent BHK164. 

### 2.3. Antifoam Studies

TubeSpin cultures were inoculated with 1.0 × 10^6^ cells/mL and resuspended in 100% fresh BHK200 media supplemented with 10 µL (0.03%), 100 µL (0.3%), 500 µL (1.7%) or 1 mL (3.3%) of antifoam (FoamAway Irradiated AOF [animal origin-free] Antifoaming Agent, Thermo Fisher Scientific, Waltham, MA, USA). The pH was adjusted to 7.5 before infection if necessary. Cells were then infected with FMDV O SAU/18/2015 at a multiplicity of infection (MOI) of 0.01. An uninfected negative control and an infected positive control with standard conditions (no antifoam) were also included. Cell controls without any addition of virus but with the addition of antifoam were cultured to test any adverse effects of the reagent on cell viability. The experiments were performed three times independently.

### 2.4. Cell Density Experiments

#### 2.4.1. Spin Tube Experiments 

TubeSpin Bioreactors-50 (TPP) with a working volume of 30 mL were inoculated with 0.5 × 10^6^ cells/mL, 1.0 × 10^6^ cells/mL or 1.5 × 10^6^ cells/mL. The cells were pelleted at 290× *g* for 5 min and resuspended in 100% fresh media. The cultures were placed in the shaker incubator overnight to reach cell densities of 1 × 10^6^, 2 × 10^6^ and 3 × 10^6^ cells/mL, respectively. Then, the cells were pelleted again and a media exchange of 100% or an addition of 30% fresh medium was performed. After resuspension of the cells, the pH was adjusted to 7.5 using 1M sodium hydroxide if necessary and the cells were then infected with FMDV A_24_ Cruzeiro or O SAU/18/2015. Cells were infected at an MOI of 0.01, and the virus was harvested after 20 h of incubation. An uninfected negative control was also included. In total, three tubes per density were prepared: one tube with virus and 30% fresh medium, one tube with virus and 100% media exchange, and one tube with no virus and 30% fresh medium. Virus samples were stored at −70 °C, then thawed for further processing. Cell debris was removed by centrifugation at 3200× *g* for 10 min at 4 °C. The experiment was performed three times independently for each of the culture media BHK200, BHK300B and BHK300G.

#### 2.4.2. Bioreactor Experiments 

Bioreactor experiments were performed using Mobius® 3L single-use bioreactors (Merck KGaA, Darmstadt, Germany) with a working volume of two liters. During the process, pH was controlled at 7.5, whereas dissolved carbon dioxide was controlled at 5% air saturation by sparging with air and/or carbon dioxide. In order to prevent foaming, FoamAway Irradiated AOF Antifoaming Agent (Thermo Fisher Scientific) was added at a maximal volume of 3 mL. Temperature and agitation were set to 37 °C and 140 rpm, respectively. Cell density was adjusted to 3 × 10^6^ cells/mL before infection. For the 100% media exchange, cells were pelleted at 290× *g* for 5 min and resuspended in fresh media. For the addition of 30% fresh media, cells were grown in 70% of the final volume and 30% fresh media was added before infection. When the pH, temperature and CO_2_ set points were reached, cells were infected with FMDV O SAU/18/2015 at an MOI of 0.01. The virus was harvested after 20 h of incubation. The experiments were performed two times independently for each cell culture media. 

### 2.5. Effect of Conditioned Culture Media on Virus Propagation 

For the experiments concerning the effect of conditioned (spent) medium on virus propagation, spent medium of the 100% media exchange bioreactor experiments was used. The spent medium was collected before virus infection. TubeSpin Bioreactors-50 with 30 mL liquid volume were inoculated at a density of 1.0 × 10^6^ cells/mL or 3.0 × 10^6^ cells/mL. Six ratios of spent medium were tested, namely 0% (fresh medium), 10%, 30%, 50%, 70% and 100%. The pH was adjusted to 7.5 and cells were infected with FMDV O SAU/18/2015 at an MOI of 0.01. Virus was harvested after 20 h of incubation. The experiments were performed three times independently. 

### 2.6. Batch Culture Experiments

Batch experiments were performed in duplicates, three times individually, with a seeding density of 0.5 × 10^6^ cells/mL for cells in BHK200 and 0.6 × 10^6^ cells/mL for cells in BHK300B and G. After one or two days, when cells reached a density of 1 × 10^6^ and accordingly 3 × 10^6^ cells/mL, either a full media exchange was performed or 30% of fresh media was added. Viable cell density (VCD) and viability in percent were measured daily up to four days after cell inoculation and were evaluated by a dye exclusion method with the Vi-CELL XR 2.04 (Beckman Coulter, Fullerton, CA, USA). The cell culture parameters acetate, calcium, iron, glucose, glutamine, glutamate, lactate, LDH, magnesium, ammonia, phosphate and pyruvate were determined for every day as well as before and after media exchange using colorimetric or turbidometric methods with the CEDEX Bio HT (Roche, Mannheim, Germany). Osmolarity was determined by the freeze point method using an Osmomat (Gonotech, Berlin, Germany). The amino acid analysis was performed using a pre-column derivatization employing the AccQ Tag Ultra reagent kit. Derivatization, chromatography and data analysis were performed according to the supplier recommendations (Waters, Milford, MA, USA). These analyses were carried out at the laboratory of Merck KGaA. Due to the restrictions for work with FMDV, they had to be performed separately from the infection experiments. 

### 2.7. Calculations

The cell-specific calculations were performed according to the equations given by [14]:

#### 2.7.1. Specific Growth Rate: *μ* (h^−1^)

$$\mu =\frac{\ln X_{n}-\ln X_{n-1}}{t_{n}-t_{n-1}}$$

*X*: VCD (per mL); *t*: time points of sampling (hour); *n* and *n* − 1: two consecutive sampling points.

#### 2.7.2. Cell Division Number (Cd):

$$\mathrm{Cd}=\log_{2}\;\frac{\text{Cell density level at the end of the culture}}{\text{Cell density level at inoculation}}$$

### 2.8. Statistical Analysis

The data were analyzed using GraphPad Prism version 07.04 for Windows (GraphPad Software, La Jolla, CA, USA). Ordinary one-way Analysis of Variance was done with Tukey’s post test to evaluate differences between treatment groups. Statistical significance was declared at *p*-values < 0.01.

## 3. Results

### 3.1. Antifoam Studies 

**Figure 1:**
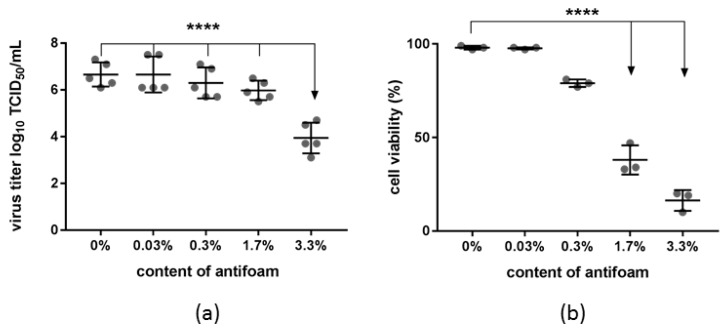
Impact of antifoam in the cell culture media on viral yield in infected cultures and cell viability in the absence of infection. Antifoam was added to the media at a final concentration of 0.03, 0.3, 1.7 or 3.3% (*v/v*). A culture with no additives served as a control. (**a**) Cells at a density of 1 × 10^6^ cells/mL were infected with Foot-and-mouth disease virus (FMDV) O SAU/18/2018 at a multiplicity of infection (MOI) of 0.01 and the viral harvest at 20 hpi is shown as log_10_ TCID_50_/mL. (**b**) Antifoam was added to non-infected cells (1 × 10^6^ cells/mL) and cell viability was measured after 20 h of culturing. Experiments were performed three times independently. Significance code: “****” *p* ≤ 0.0001.

The addition of defoaming agents is a common way to prevent the formation of foam inside a bioreactor. To exclude the possibility that the addition of antifoam negatively affects viral infectivity, experiments were conducted using an antifoaming reagent with 30% simethicone. Small volumes of antifoam were added, resulting in percentages of 0.03, 0.3, 1.7 and 3.3% of antifoam relative to the total volume used for the experiment. Up to 1.7% of antifoam in the cell culture media had no significant impact on the viral yield, even though a decreasing trend was evident above a concentration of 0.3%. Viral titers were around 6.4 ± 0.6 log_10_ TCID_50_ per mL. A significant decrease in viral titer down to 3.9 ± 0.7 log_10_ TCID_50_ per mL was seen when the content of antifoam reached 3.3% of the total volume (*p* < 0.0001, Figure 1a). However, the cultivation of uninfected cells with added antifoam revealed the heavy impact of antifoam alone on cellular viability, resulting in a decrease in viability already at an antifoam concentration of 0.3% and a significant loss of viability down to 38 ± 8% and 16 ± 6% at concentrations of 1.7% and 3.3%, respectively (*p* < 0.0001, Figure 1b).

### 3.2. Cell Density Effects

**Figure 2:**
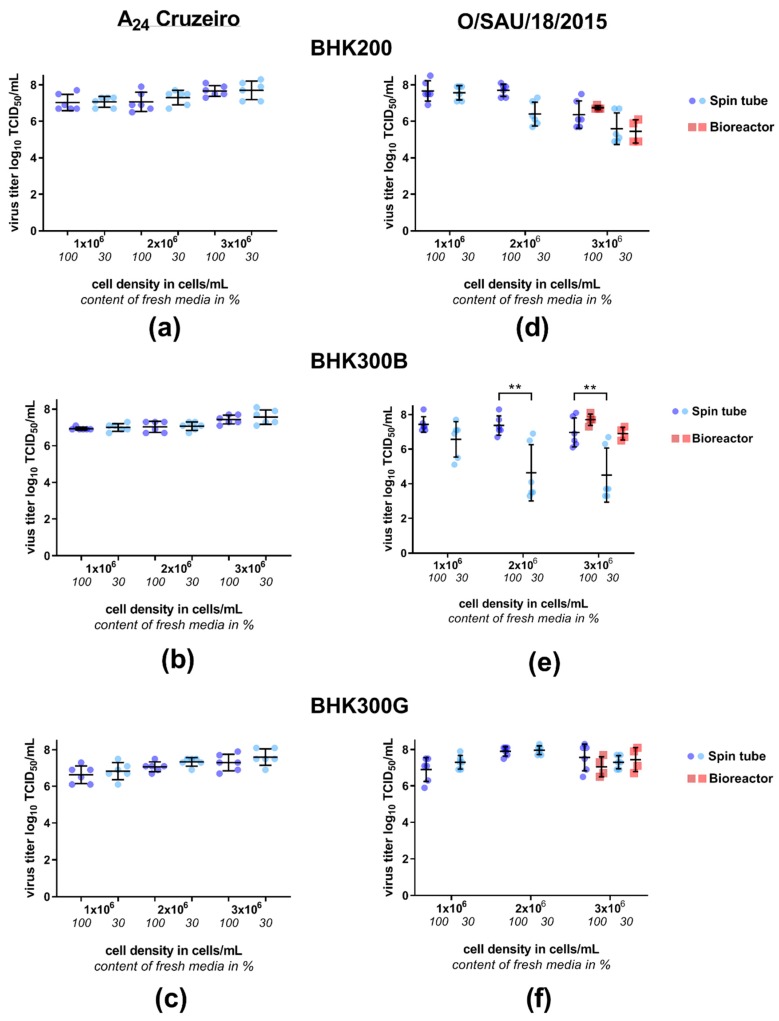
Cell density experiments in spin tubes and 3L-single-use bioreactors with different cell culture media for cell growth. Cells were grown in (**a**,**d**) the animal-component-free (ACF) media BHK200 or in the CDM (**b**,**e**) BHK300B and (**c**,**f**) BHK300G in three different cell densities (1 × 10^6^/2 × 10^6^/3 × 10^6^ cells/mL). A media exchange of either 100% or addition of 30% fresh media was performed before infection with FMDV A24 Cruzeiro (panels **a**–**c**) or O/SAU/18/2015 (panels **d**–**f**) at a MOI of 0.01. The final virus titers in log_10_ TCID_50_/mL of the spin tube experiments are colored in blue, while the results of the bioreactor runs are shaded in red. Significance code: “**” *p* ≤ 0.01.

To examine the impact of cell density on the viral titer, two virus isolates, A_24_ Cruzeiro and O/SAU/18/2015, were incubated at three different cell densities (1 × 10^6^, 2 × 10^6^ and 3 × 10^6^ cells/mL) in combination with two different media supplementation strategies (100% media exchange or addition of 30% fresh media). Tested were three different cell culture media, a serum and animal component free media (BHK200) and two chemically defined cell culture media. The different test conditions had no influence on the viability of non-infected cells (Figure S3). No impact on the viral yield was seen for the laboratory-adapted serotype A isolate A_24_ Cruzeiro between the conditions of 30% and 100% fresh media in any of the tested culture media. Independent of the culture media, the viral titer ranged roughly between 6.6 and 7.6 log_10_ TCID_50_ per mL with an increasing trend, albeit without statistical significance, towards a higher viral yield with increasing cell density (Figure 2, panel A–C). For the serotype O strain, distinct differences in viral titer were visible depending on cell culture media and media exchange before infection. The chemically defined media “BHK300G” did not show any significant differences in viral titer, independent of the applied condition. Viral titers were between 6.9 and 8.0 log_10_ TCID_50_ per mL, also exhibiting a low variance in titer (Figure 2, panel F). By contrast, the second chemically defined cell culture media “BHK300B” displayed large drops in titer with 30% media supplementation. While a cell density of 1 × 10^6^ cells/mL had no influence on viral growth, cell densities of 2 × 10^6^ and 3 × 10^6^ cells/mL led to a decrease in the viral titer, if only 30% of fresh media were added to the culture before infection (*p* = 0.0069; *p* = 0.0023, Figure 2, panel E). With the animal-component-free media BHK200, there was no difference between infections performed with the two different media supplementation strategies at low cell densities of 1 × 10^6^ cells/mL. With increasing cell density, the viral titer dropped for 30% media supplementation compared to the 100% media replacement, although these differences were not statistically significant. Furthermore, the variability of the final titers between experiments increased up to a difference of ±1.0 log_10_ TCID_50_ per mL for cell densities of 3 × 10^6^ cells/mL (Figure 2, panel D). 

Based on these results, the large-scale bioreactor experiments were only performed with a cell density of 3 × 10^6^ cells/mL and FMDV serotype O, given that this combination gave the largest discrepancies in the viral titers between the different culture media and the different supplementation strategies. As expected, no differences in viral titer between the different media conditions were visible in the bioreactors when growing the cells in BHK300G (Figure 2, panel F, red data points). For BHK200, the same trend that was seen in the spin tube experiments, namely a decrease in the viral yield if no 100% media refreshment had been performed, was also evident in the bioreactor experiments (Figure 2, panel D, red data points). The viral growth in BHK300B benefitted from the controlled conditions of a bioreactor. Although important variables such as pH were within range also at the end of the spin tube experiments, a presently undetermined variable seems to be better controlled during the bioreactor runs, resulting in significantly higher viral titers compared to the spin tube experiments. In addition, no statistically significant difference in the viral yield was evident any longer between a 30% and a 100% media exchange before infection (Figure 2, panel E, red data points).

### 3.3. Conditioned Media

**Figure 3:**
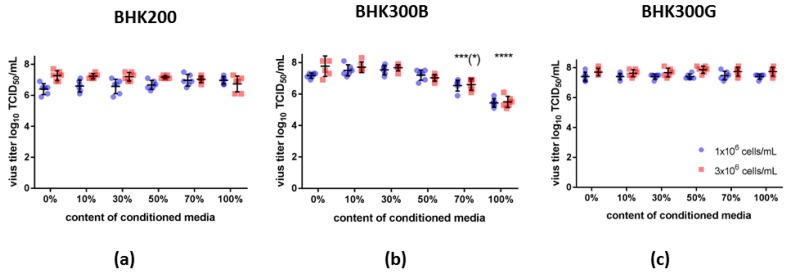
Virus growth in cell culture media with different contents of conditioned media in spin tubes. Cells were grown in the (**a**) ACF media BHK200 and in the chemically defined media (CDM) (**b**) BHK300B and (**c**) BHK300G in two different cell densities (1 × 10^6^ cells/mL: blue; 3 × 10^6^ cells/mL: red). Different percentages of 0%, 10%, 30%, 50%, 70% and 100% conditioned media were applied before viral infection with FMDV O/SAU/18/2015 at a MOI of 0.01. Significance code: “****” *p* ≤ 0.0001, “***” *p* ≤ 0.001.

Possible explanations for the occurrence of the observed cell density effects include an accumulation of inhibitory factors or a limitation of available nutrients in the cell culture media. Therefore, experiments were conducted with different contents of pre-conditioned (spent) media, either at a cell density of 1 × 10^6^ or 3 × 10^6^ cells/mL. For all cell culture media, higher cell densities at infection usually led to slightly higher viral titers. For the BHK200 media, the viral titers remained relatively stable at 6.7 ± 0.4 log_10_ TCID_50_ per mL for cell densities of 1 × 10^6^ cells/mL and 7.1 ± 0.3 log_10_ TCID_50_ per mL for a cell density of 3 × 10^6^ cells/mL. This could indicate a decreasing trend for the viral titer and an increase in variability for virus growth in 100% conditioned media and a cell density of 3 × 10^6^ cells/mL (Figure 3, panel A). The viral yield from cultivation in the BHK300B media remained stable at 7.4 ± 0.3 log_10_ TCID_50_ per mL at a cell density of 1 × 10^6^ cells/mL up to a volume of 50% of conditioned media. For 70% and 100% spent media at the time of infection, the viral titer dropped significantly by one to two log_10_ TCID_50_ per mL. The same was evident at a cell density of 3 × 10^6^ cells/mL, although the decrease in viral titer already started at 50% spent media (Figure 3, panel B; all significant differences and the exact data assignment are shown in Supplementary Table S2). Similar to the earlier experiments, viral growth in the BHK300G media was not influenced by the content of conditioned media. The viral titer remained stable at 7.4 ± 0.2 log_10_ TCID_50_ per mL for a cell density of 1 × 10^6^ and 7.7 ± 0.2 log_10_ TCID_50_ per mL for a cell density of 3 × 10^6^ cells/mL (Figure 3, panel C). 

**Figure 4:**
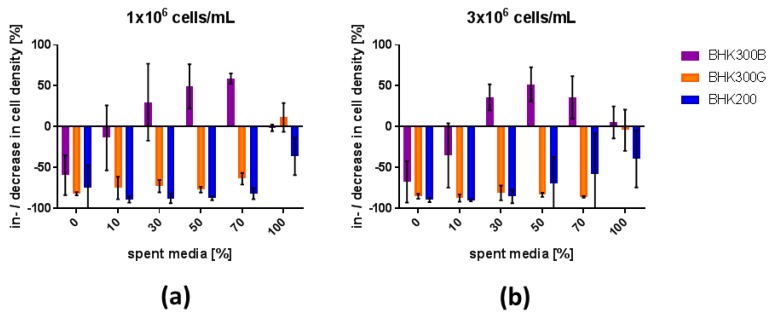
Increase or decrease of the cell density relative to the starting cell density. Increase or decrease in cell density after 20 h of viral infection at a cell density of (**a**) 1 × 10^6^ cells/mL or (**b**) 3 × 10^6^ cells/mL. Values were calculated from the values recorded in the experiment described above (Figure 3).

An evaluation of the cell density during the pre-conditioned media experiments revealed an expected decrease in the cell density at 20 hpi for BHK300G and BHK200 up to a content of 70% of spent media. By contrast, cell density in BHK300B was more variable, and rather than decreasing as virus-infected cells are lysed, the cell density even increased at 20 hpi. For viral infections in 100% spent media, cell densities stagnated but BHK300G still performed best (Figure 4).

### 3.4. Batch Culture Experiments

**Figure 5:**
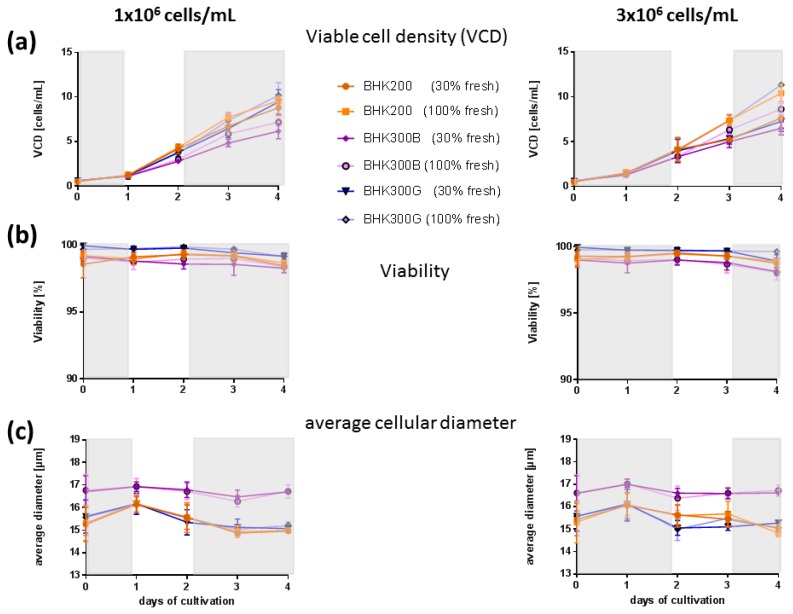
Cellular phenotype during batch cultivation. Cellular parameters such as (**a**) viable cell density (VCD) in cells/mL, (**b**) percent viability and (**c**) average diameter in µm were evaluated for BHK200 (orange), BHK300B (violet) and BHK300G (blue). Highlighted in white is the period of time in which viral infection and cellular virus production would take place at cell densities of 1 × 10^6^ cells/mL or 3 × 10^6^ cells/mL.

Batch culture experiments were performed to answer the question whether nutrient limitations restrict the cell-specific productivity or if metabolic waste products accumulate in the media that could inhibit the viral infection. The data were analyzed with regard to differences in cell density and differences between the three cell culture media. The cellular parameters VCD and viability did not differ markedly between the cell culture media (Figure 5, panels A and B). The viability was always between 98% and 100% and the cell density was increasing over a period of at least four days in culture. Differences were seen in the average diameter of the cells, with the cells in BHK300B being approximately one micron larger than the cells in BHK300G and BHK200 (Figure 5, panel C). 

Calculation of the cell specific growth rate *μ* (h^−1^) and cell division number (cd) revealed no differences for any of the tested conditions between BHK200 and BHK300G, while cells in BHK300B grew significantly slower and divided significantly less than cells in BHK300G and BHK200 (*p* ≤ 0.0001). Only at a cell density of 3 × 10^6^ cells/mL, with a medium exchange of 100%, no differences were visible between all three culture media (Table 1). 

**Figure 6:**
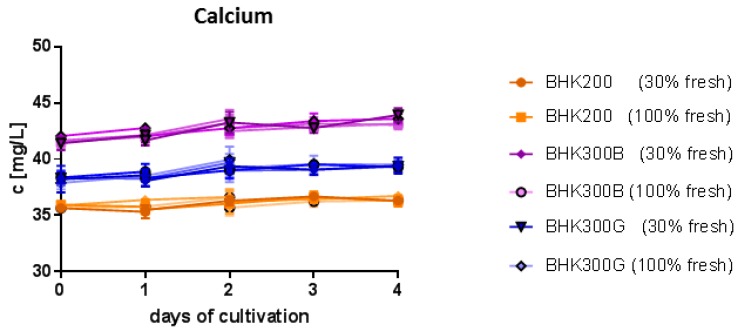
Calcium consumption of cells during batch analysis. Cells were grown in BHK200 (orange), BHK300B (violet) or BHK300G (blue) with either 100% medium supplementation or addition of 30% fresh medium.

Furthermore, the content of critical substrates such as glucose, glutamine, pyruvate, acetate and glutamate and metabolic waste products such as lactate, and ammonia were analyzed during batch culture. The mineral nutrients calcium, magnesium, phosphate and the trace element iron were examined as well as lactate dehydrogenase (LDH) enzyme, an indicator of cellular damage. Because virus propagation was most obviously affected in the BHK300B media, the comparison was focused on the differences between BHK300B and BHK200/300G. Most striking was the difference in the calcium concentration (highest in BHK300B and lowest in BHK200, Figure 6), while none of the other substrates differed between BHK300B on the one hand and BHK200 or BHK300G on the other, nor between the different cell densities (see Supplementary Figure S1). In addition, all nutrients were within the normal range at the time points of a potential infection and no waste product increased towards a critical limit for all three culture media. 

The analysis of the amino acids in the three different media revealed no difference for serine, glutamine, glycine, glutamate, cysteine, aspartate, alanine, cysteine (see Supplementary Figure S2.1). For the remaining amino acids, a trend in concentration was obvious with highest content in BHK300B, followed by BHK300G and BHK200 last (see Supplementary Figure S2.2). This difference was very pronounced for histidine, threonine, proline and tryptophane (see Supplementary Figure S2.3). 

## 4. Discussion

The global control and eradication of FMD is a priority of both the OIE and the FAO [19]. Vaccination is one of the key elements to reach this goal. This study intended to promote the production of better and more cost-effective FMD vaccines by investigating the influence of different cell densities at the time point of infection and the use of cell culture media free of serum and other animal-derived components. Experiments were conducted in spinner tubes with a working volume of 30 mL and in stirred tank bioreactors with a working volume of two liters. One problem when launching bioreactors is the formation of foam inside the reactor. Foam originates from stirring, sparging of gas into the liquid and from dead cells and it is a high mechanical burden for viable cells [20,21]. Chemical defoamer interacts with cell membranes [22] and it is therefore possible that antifoaming agents interfere with cell-virus interactions. A simethicone-based defoamer was used to evaluate the influence of a chemical antifoam reagent. Simethicone is an inert chemical substance that is harmless to cells [22] and broadly used in vaccine production [23]. Nevertheless, cell viability in the small-scale experiments was clearly influenced by the addition of large quantities of antifoam. Presumably resulting from the low cellular viability, a significant decrease in the viral yield at a concentration of 3.3% antifoam in the culture media was seen. Therefore, during the bioreactor experiments, antifoam was only added to the culture dropwise and in minimal concentration (less than 3 mL for a culture volume of 2 L), only as much as necessary to dissolve the foam that was present. A single application of antifoam was sufficient to prevent foam formation during the whole experiment. Compared to the capacity of the used bioreactor, 3.3% corresponds to 66 mL of antifoam. An influence of the used defoamer on the outcome of our experiments is therefore unlikely. Although the performance of defoaming agents is always system-specific and dependent on several factors such as culture medium and aeration [20,22], the fact that chemical antifoam reagents can interfere with cell survival and as a result impede FMDV infection and virus production should be kept in mind. 

Cell density effects are observed in many virus production systems [11,12] and appear to be a concern for FMDV, too. Commonly accepted explanations for these effects are the limitation of key nutrients, an inhibition through byproducts or an arrest of the cell cycle at high cell densities that diminishes the cell-specific productivity [11,24,25]. Batch experiments have been performed to evaluate the culture environment at the time of virus infection. Observation of the cultures for four days revealed that cell densities of 5 to 9 × 10^6^ cells/mL at a viability of 97% and higher are possible depending on the culture media. For all culture media and both tested cell densities, the cells are still in the exponential growth phase at the time of infection. Lactate dehydrogenase, an indicator of cellular damage, remained below the detection limit until day 3, and only stared to increase between days 3 and 4 of cell cultivation. It is therefore unlikely that cell density effects in this system are due to cell cycle arrest or restricted cell growth. A limitation of key nutrients leading to reduced cell-specific productivity is a second important factor. Critical substrates such as glucose and glutamine, minerals and trace elements were monitored during batch experiments, but none were depleted at the time point at which viral infection would take place. Because of that, we hypothesized that the reason for a diminished viral yield at high cell densities, if no complete media exchange has been performed prior to infection, is likely to be metabolic waste products that inhibit viral infection or create a disadvantageous environment for the virus. To investigate the influence of spent media on the viral yield, experiments at high and low cell density with increasing contents of spent media were performed. While the viral yield was stable between the different conditions for BHK200 and BHK300G, the viral titer in BHK300B started to decline already at 30% of conditioned media with significantly reduced viral titers from 70% of spent media upwards. In contrast to BHK200 and BHK300G, cell density in BHK300B increases over the course of infection. At 100% spent media, cells in all culture media seemed to reach a static level. It appears that the culture media rather than the cell density is the crucial factor because the results were the same for high and low cell densities. However, potentially harmful waste products such as ammonia and lactate did not exceed critical limits [26].

Among environmental parameters, the pH of the culture is the most important factor for FMDV particle stability. The FMDV capsid disassembles into pentameric subunits already at a pH of slightly under seven, with a direct correlation between loss of infectivity and acidic pH [27,28]. The influence of oxygen availability on FMDV is less clear, but it has been shown for baculoviruses that infected cells have a greater oxygen demand than non-infected cells [12]. The controlled environment of a bioreactor provides both, stable pH and sufficient oxygen supply. While virus production in BHK200 and BHK300G did not differ between the controlled bioreactor system and spin tubes, the viral yield in BHK300B was not only significantly higher in the bioreactor than in spin tubes, there was also no significant difference between the different media exchange strategies in the former. 

A loss of titer was especially obvious for virus produced in cells that grow in BHK300B. An interesting observation in this context was the larger cell size of the cells grown in BHK300B in comparison to the cells growing in BHK200 and BHK300G. The regulation of cell size is a multifactorial event with many influencing variables [29] and the specific reason why cells in one media are bigger than cells in other media is impossible to determine. However, the so-called “adder” principle proposes that large cells grow less than small cells [29]. This is in accordance with our data. Cells in BHK300B grow significantly slower than in the other media, which might lead to lower cell-specific productivity. 

The extent of cell density effects and the viral yield can be influenced by the composition of the culture media [11]. The BHK300B media is higher in calcium than the other media. Calcium is an important mineral for the binding of ligands to integrins and heparan sulfate [30,31], two important cellular receptors for FMDV [32,33,34]. While manganese and magnesium support ligand binding to integrins, calcium is a competitive inhibitor for magnesium [35] and binding of FMDV to integrin αvβ1 has been shown to be relatively inefficient at physiological concentrations of calcium and magnesium [34]. Ligand binding to heparan sulfate is also reduced with increasing concentrations of calcium [31] and at least in a parathyroid cell line the distribution and recycling of heparan sulfate depends on the calcium concentration [36]. Therefore, high extracellular calcium leads to a loss in heparan sulfate at the cellular surface and a tenfold reduced recycling of the receptor [36], which could have a negative influence on FMDV infection.

In addition, the BHK300B media contains dextran sulfate, a polyanionic sulfated polysaccharide that increases cellular growth and productivity and prevents clumping of suspension cells by an inhibition of protein binding [37]. The replication of many enveloped viruses is inhibited by the attachment of dextran sulfate to the viral capsid [38,39]. We found no studies of FMDV and dextran sulfate, but other picornaviruses such as hepatitis A virus, poliovirus and coxsackievirus were inhibited, either in their attachment to the cell or during the release of mature virions [40,41,42]. 

## 5. Conclusions 

Our results suggest the conclusion that a high cell density at the time of infection does not significantly increase the viral titer, although an increasing trend is visible for the highly cell culture-adapted viral strain A_24_ Cruzeiro. In the presence of spent media, high cell densities lead to a variable and often reduced yield, at least for the serotype O strain. The reasons for this phenomenon lie in the media composition; however, it might also be affected by the fragile relationship between the virus life cycle and cell metabolism. Some of these harmful interactions can be avoided in the stable and controlled environment of an automated stirred-tank bioreactor. 

## Figures and Tables

**Table 1:** Calculation of the cell-specific growth rate and cell division.

	Cell Density	Media Exchange	Growth Rate *μ* (h^−1^)			Cell Division Number (cd)		
			$\bar{X}$	s	N	$\bar{X}$	s	N
**BHK200**	1 × 10^6^ cells/mL	+30%	0.06	0.00	6	1.92	0.16	6
	1 × 10^6^ cells/mL	+100%	0.06	0.01	6	2.05	0.18	6
	3 × 10^6^ cells/mL	+30%	0.03	0.00	6	0.87	0.12	6
	3 × 10^6^ cells/mL	+100%	0.04	0.00	6	1.36	0.12	6
**BHK300B**	1 × 10^6^ cells/mL	+30%	0.04	0.00	6	1.47	0.10	6
	1 × 10^6^ cells/mL	+100%	0.04	0.00	6	1.54	0.07	6
	3 × 10^6^ cells/mL	+30%	0.02	0.00	6	0.83	0.16	6
	3 × 10^6^ cells/mL	+100%	0.03	0.00	6	1.13	0.10	6
**BHK300G**	1 × 10^6^ cells/mL	+30%	0.06	0.00	6	2.07	0.16	6
	1 × 10^6^ cells/mL	+100%	0.06	0.00	6	2.02	0.17	6
	3 × 10^6^ cells/mL	+30%	0.03	0.01	6	0.94	0.17	6
	3 × 10^6^ cells/mL	+100%	0.04	0.00	4	1.40	0.07	4

$\bar{X}$ arithmetic mean; s standard deviation; N number of values.

## Supplementary Materials

The following are available online at https://www.mdpi.com/1999-4915/11/6/511/s1, Figure S1: Results of cell culture parameter analysis of cell culture during batch culture propagation; Figure S2: Results of amino acid analysis for cell culture during batch culture propagation.; Figure S3: Cell viability in cell density experiments 20 hpi. Table S1: Overview about the samples used in this study, their origin and passage history, Table S2.1: Statistics and significances for conditioned media testing in BHK300B with a cell density of 1 × 10^6^ cells/mL, Table S2.2: Statistics and significances for conditioned media testing in BHK300B with a cell density of 3 × 10^6^ cells/mL.
