# Supplementary figures and images for: Cell Density Effects in Different Cell Culture Media and Their Impact on the Propagation of Foot-And-Mouth Disease Virus
Source: Viruses. 2019 Jun 4;11(6):511. doi: 10.3390/v11060511 (PMC6631978; doi:10.3390/v11060511)

## Slide 1
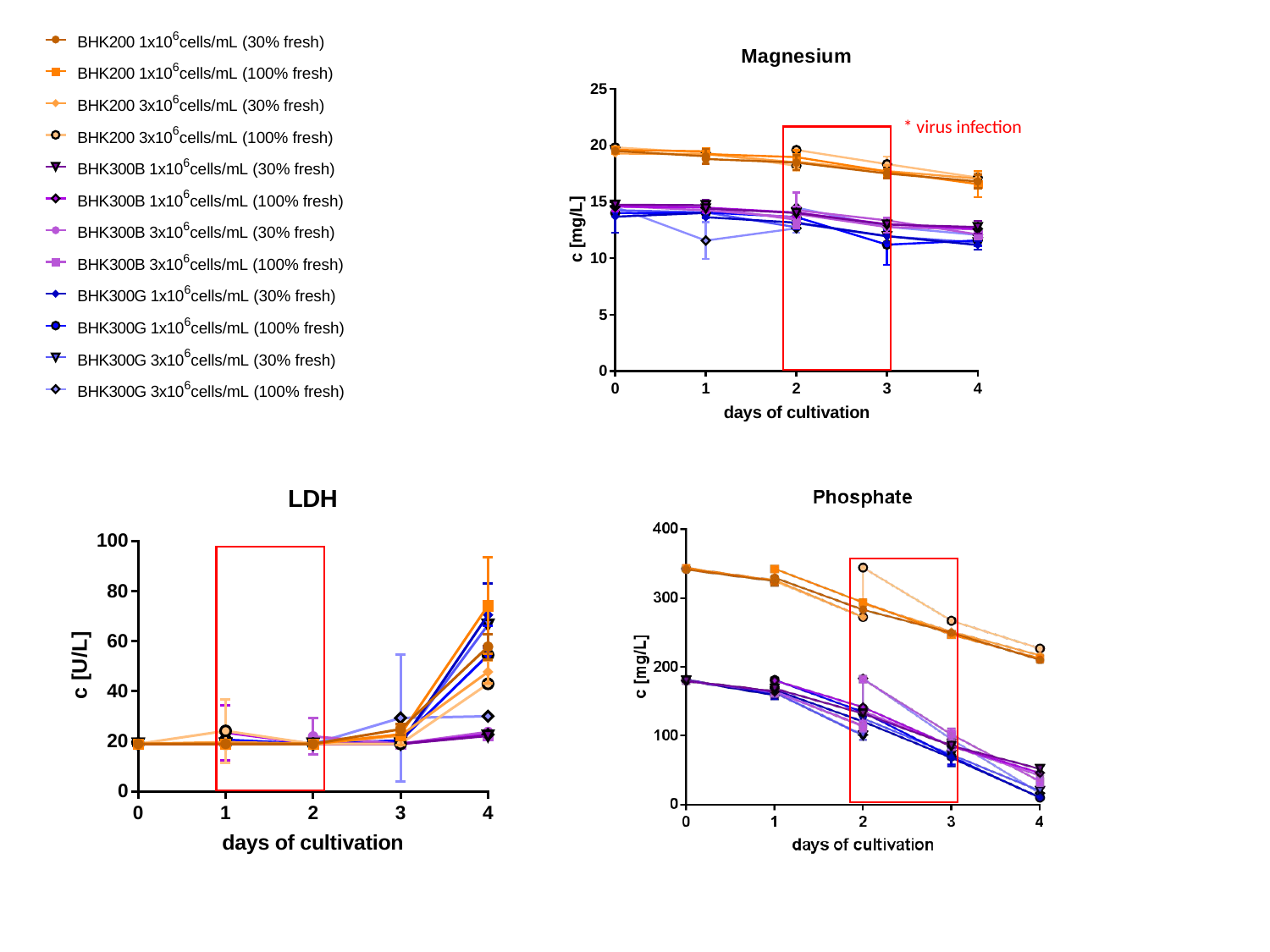

* virus infection

## Slide 2
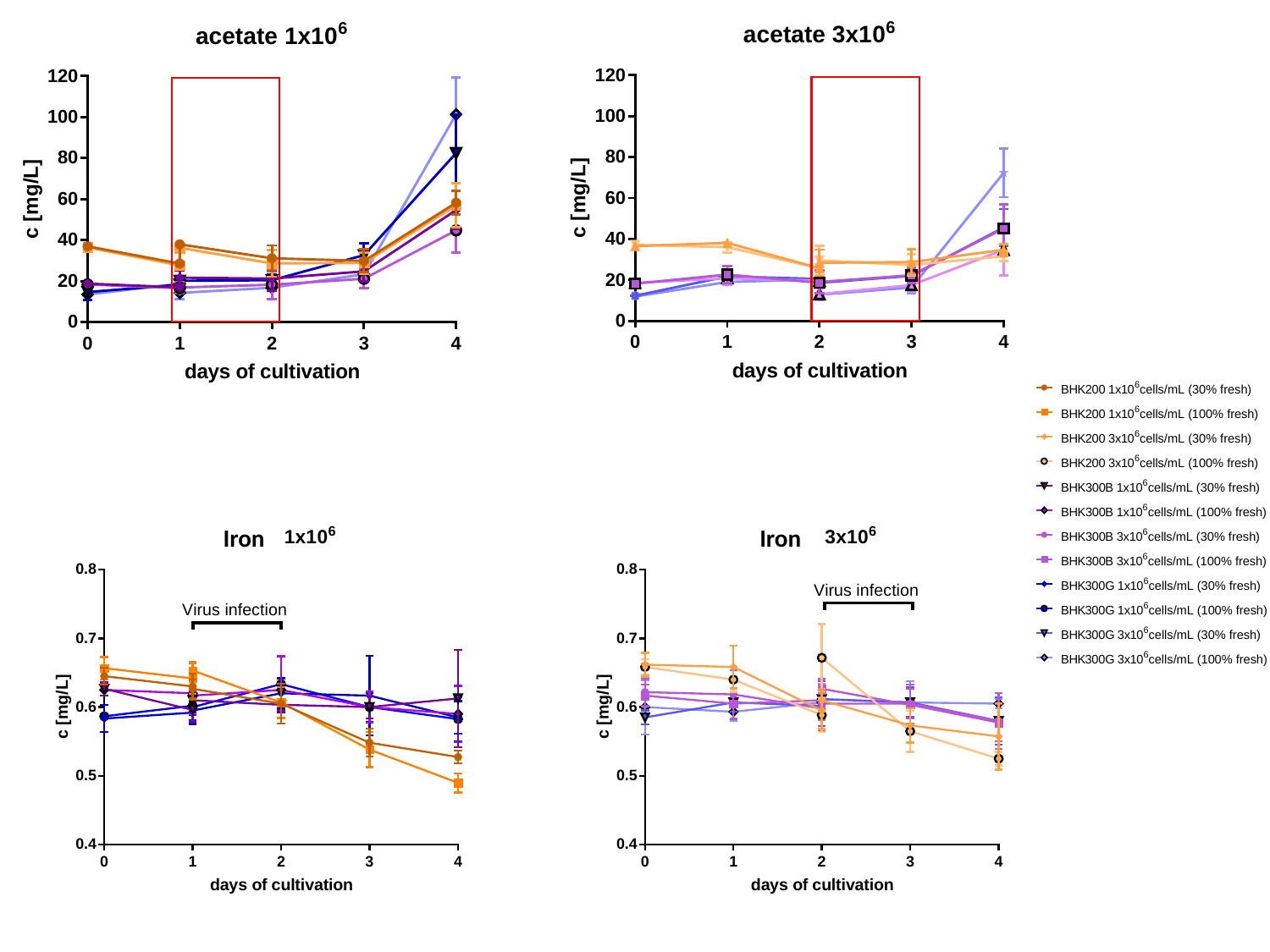

Iron
Iron

## Slide 3
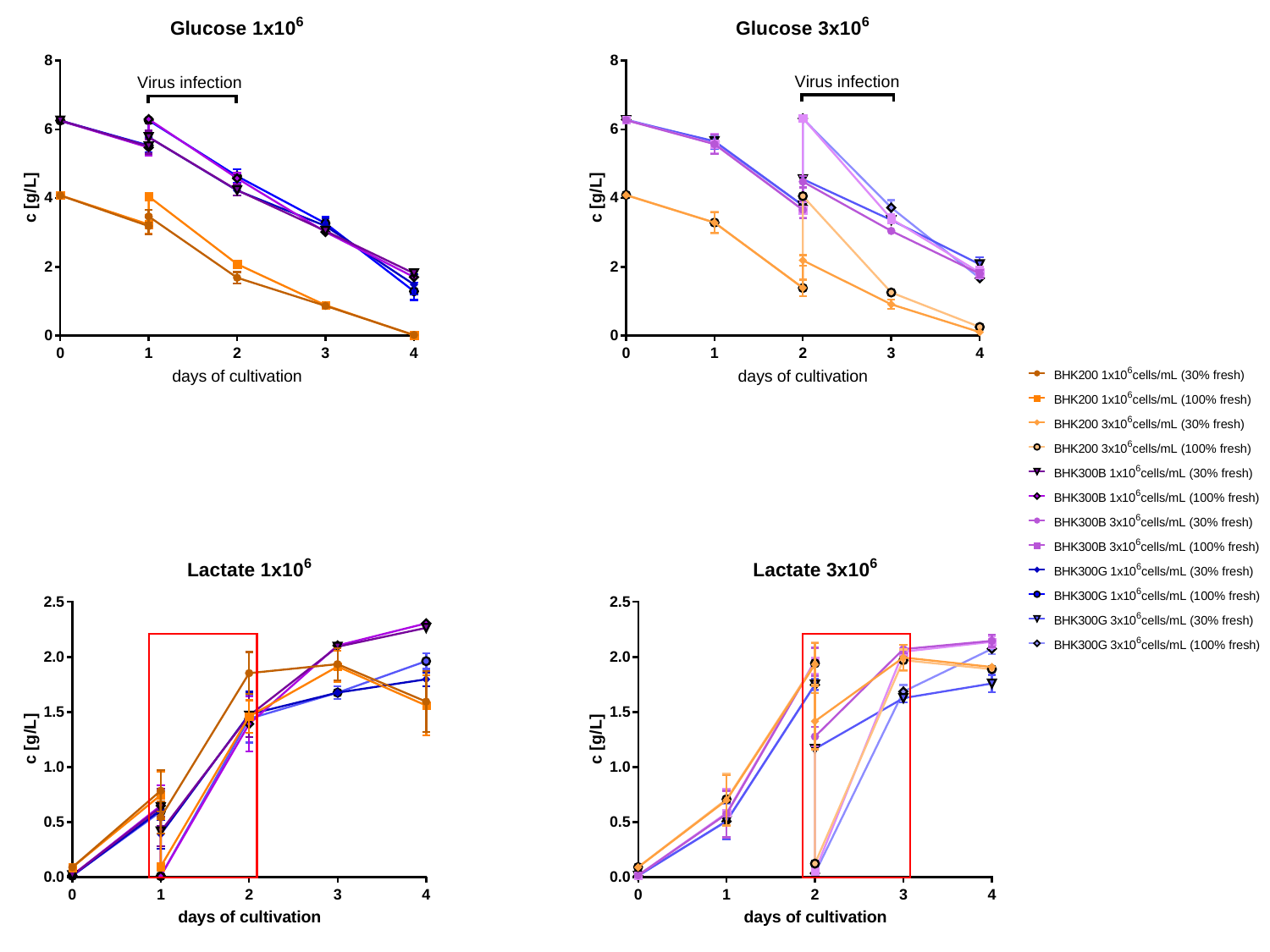

## Slide 4
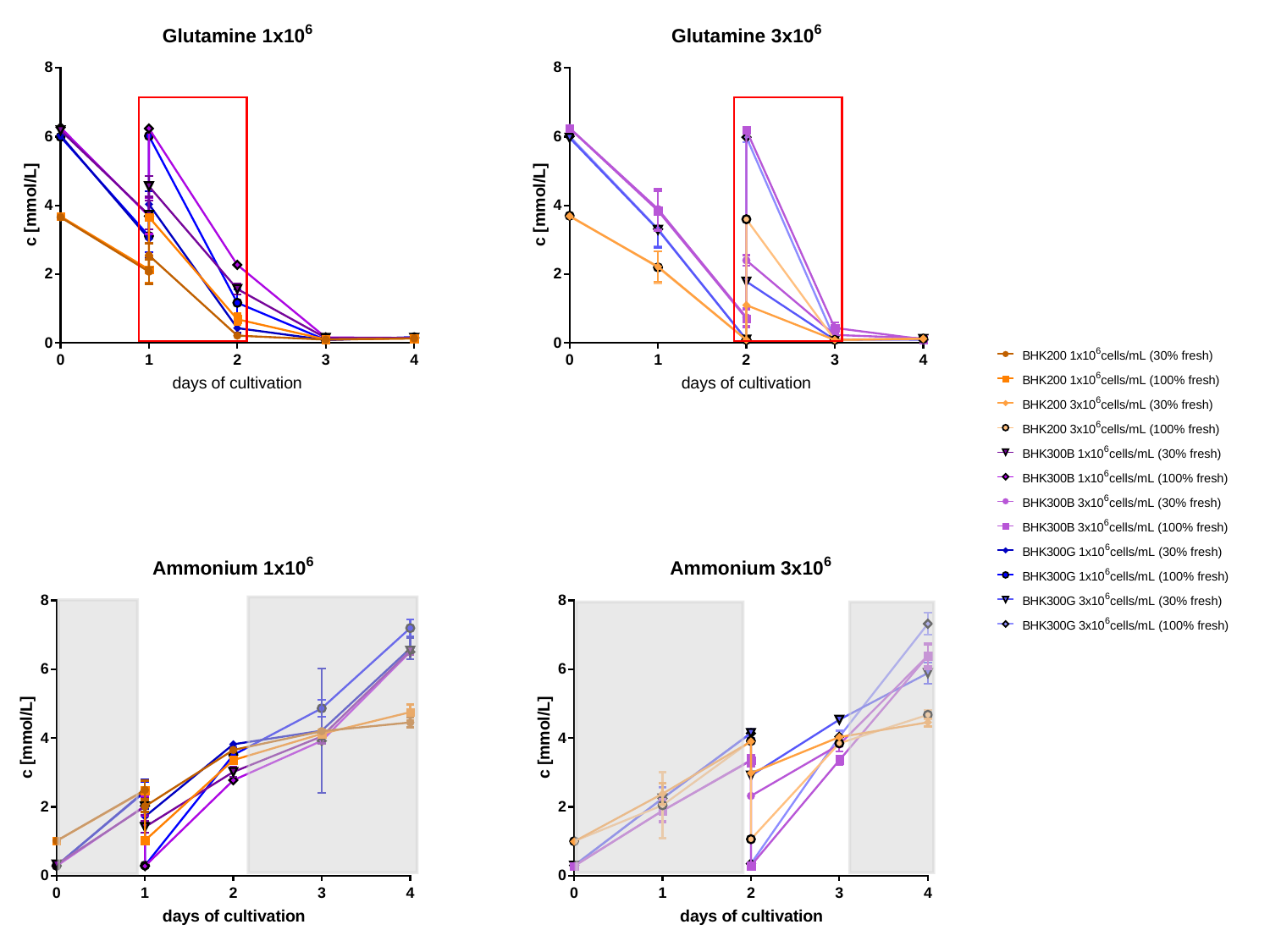

## Slide 5
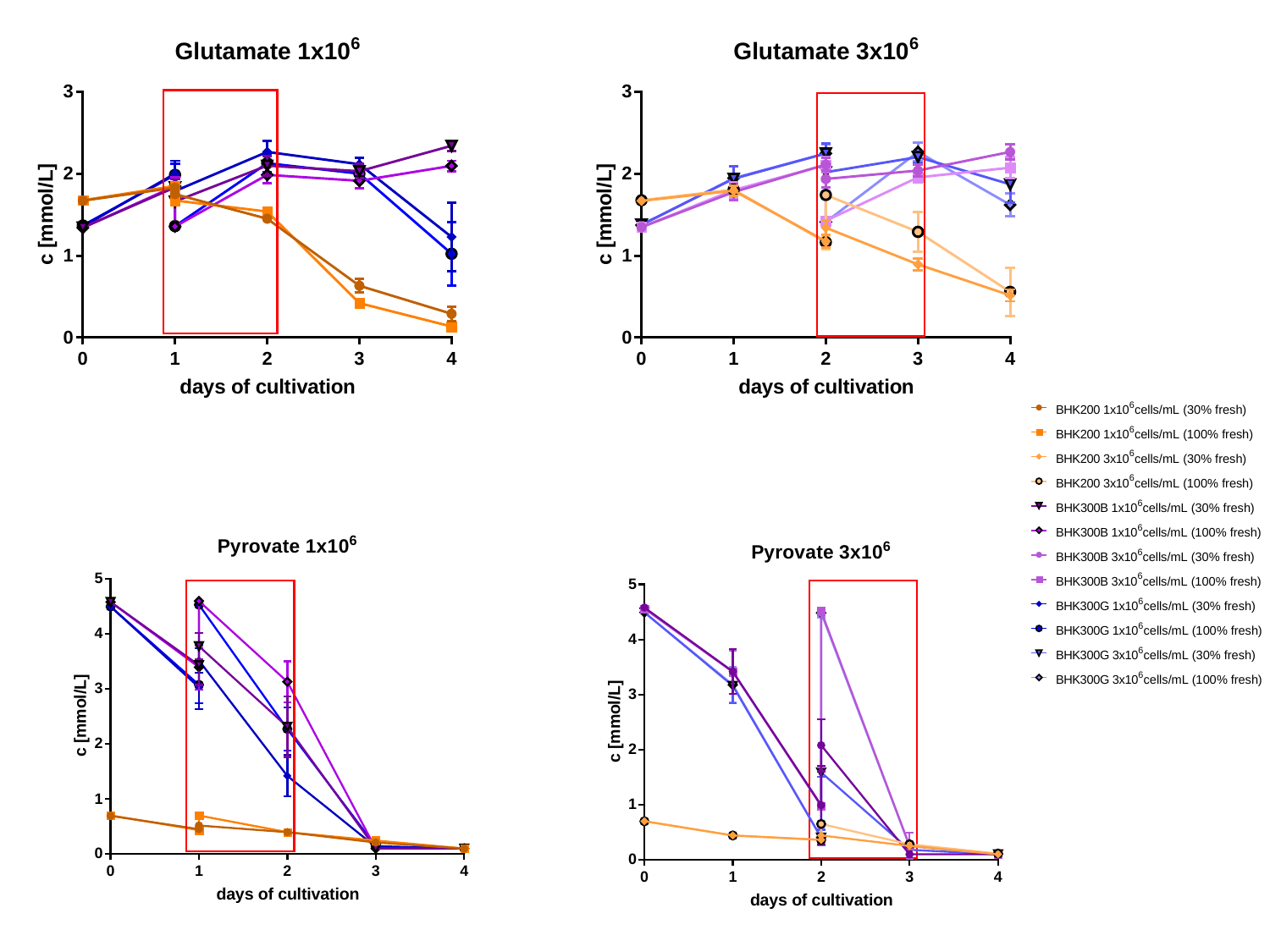

Supplement: Supplementary file 1 [file viruses-11-00511-s001.zip › Fig S1.pptx]

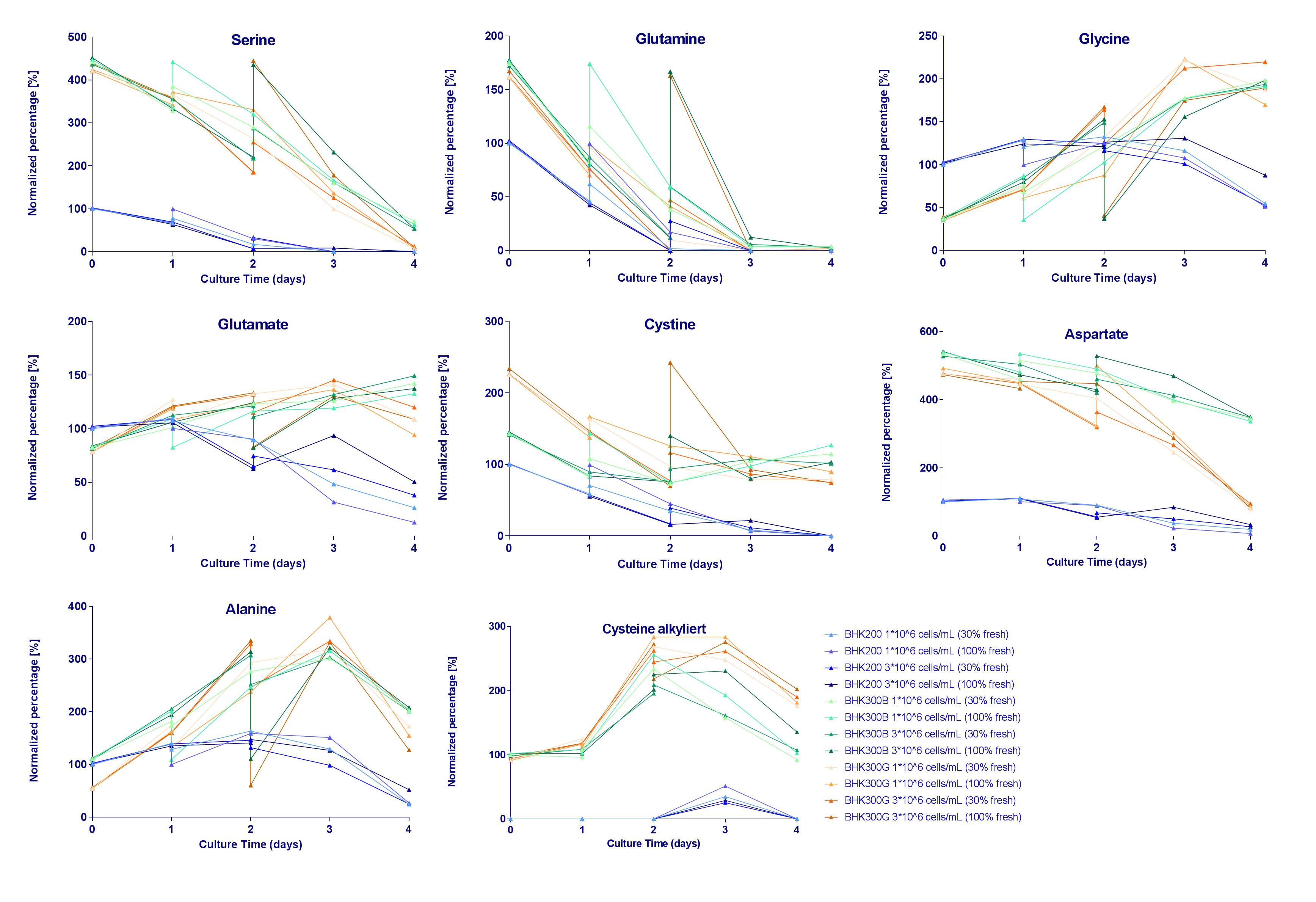

Supplement: Supplementary file 1 [file viruses-11-00511-s001.zip › Fig S2.1.tif]

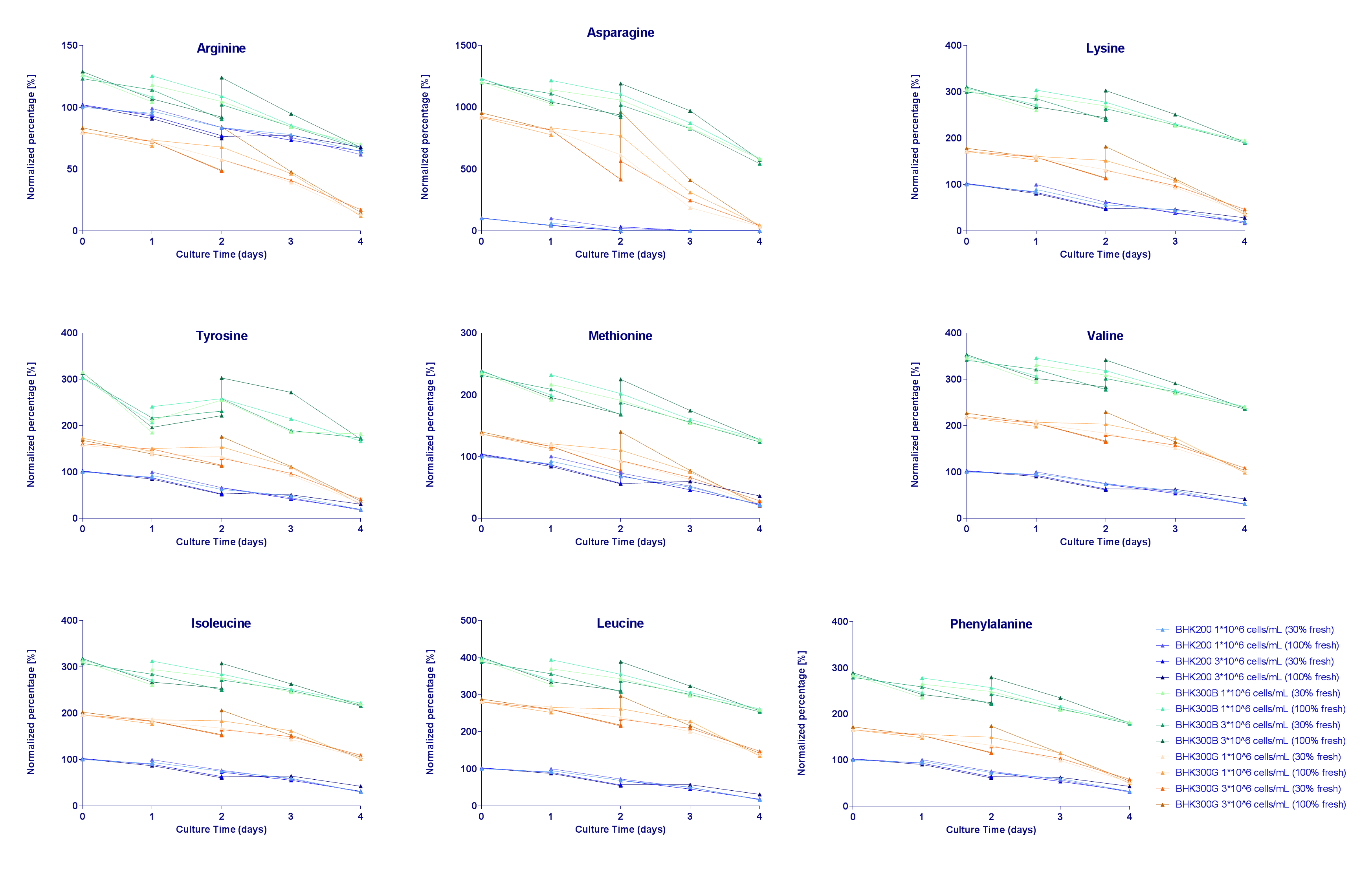

Supplement: Supplementary file 1 [file viruses-11-00511-s001.zip › Fig S2.2.tif]

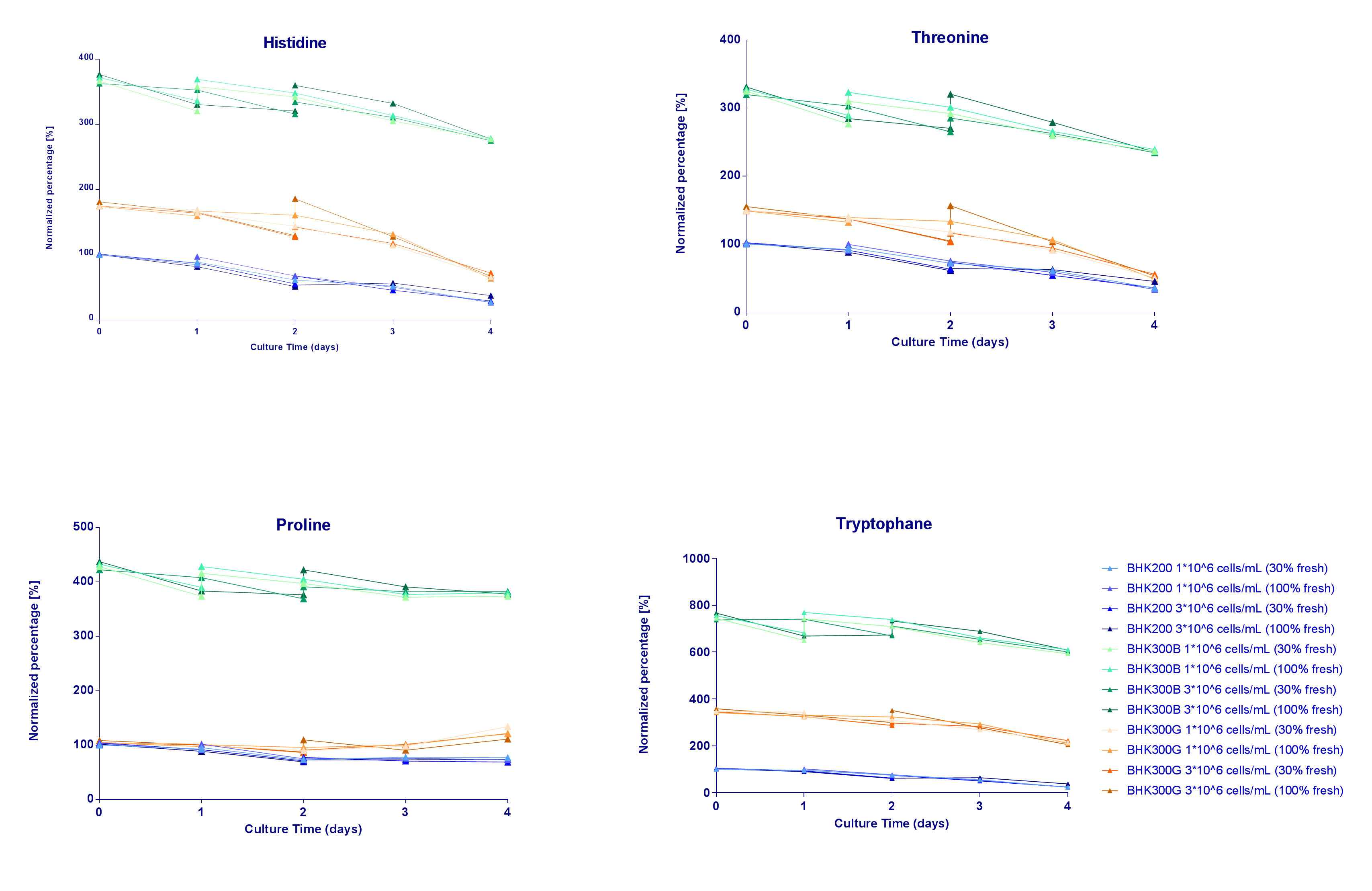

Supplement: Supplementary file 1 [file viruses-11-00511-s001.zip › Fig S2.3.tif]

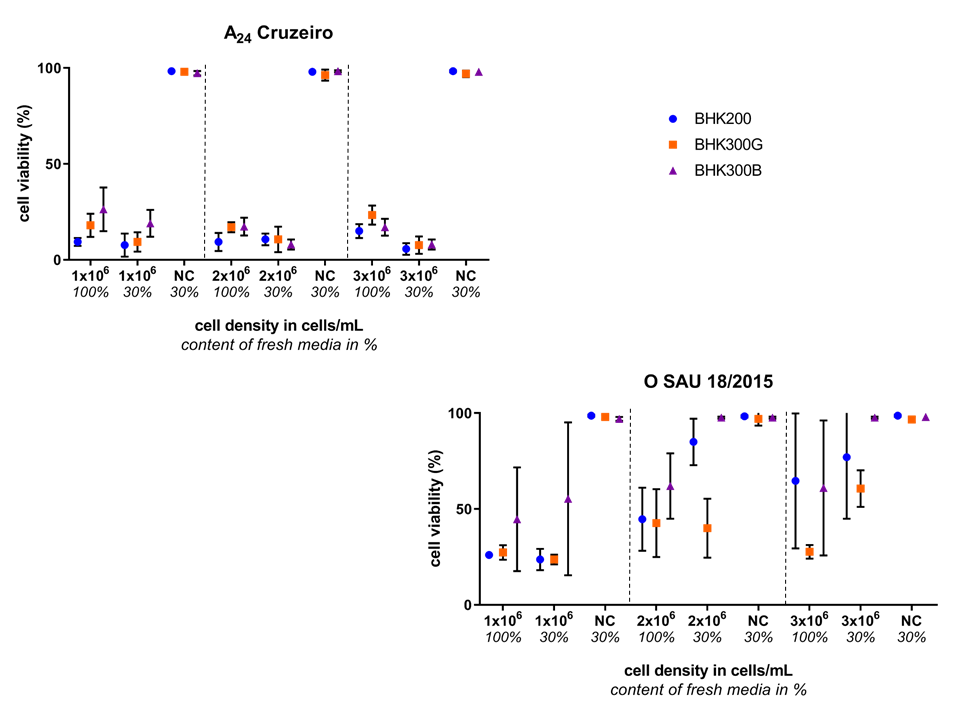

Supplement: Supplementary file 1 [file viruses-11-00511-s001.zip › Fig S3.tif]
